# Supplementary material for: H2O2 and Engrailed 2 paracrine activity synergize to shape the zebrafish optic tectum
Source: Commun Biol. 2020 Sep 29;3:536. doi: 10.1038/s42003-020-01268-7 (PMC7524761; doi:10.1038/s42003-020-01268-7)
Supplement: Supplementary file 1 — Supplementary Information [file 42003_2020_1268_MOESM1_ESM.docx]

Supplementary Materials for

**H_2_O_2_ and Engrailed 2 paracrine activity synergize to shape the Zebrafish optic tectum**

Irène Amblard, Marion Thauvin, Christine Rampon, Isabelle Queguiner, Valeriy V. Pak, Vsevolod Belousov, Alain Prochiantz, Michel Volovitch, Alain Joliot, Sophie Vriz

Correspondence to: [vriz@univ-paris-diderot.fr](mailto:vriz@univ-paris-diderot.fr) and [alain.joliot@college-de-france.fr](mailto:alain.joliot@college-de-france.fr)

**This PDF file includes:**

Supplementary Figure S1 to S5

SupplementaryTables S1 to S4

Abbreviations used in Supplementary Tables S2 and S3

References

**Supplementary Figure S1. Eng2 distribution at 24 hpf**

**
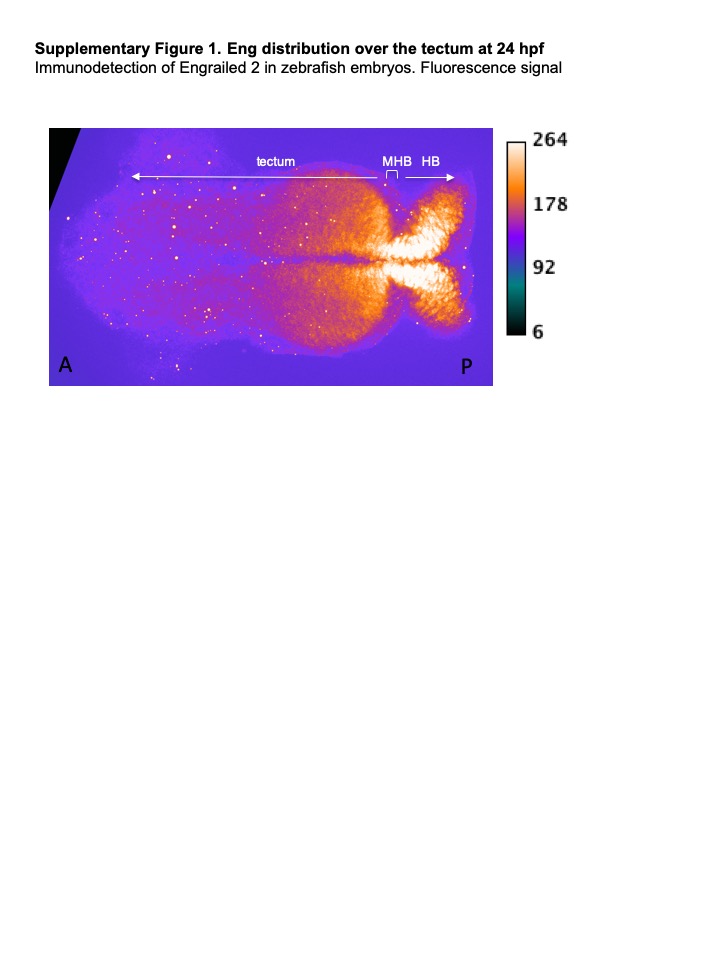
**

Immunodetection of Eng2a and Eng2b in zebrafish embryos corresponding to the image in Fig 1a without DAPI staining. Protein gradient quantified in Figure 1b is clearly visible from the MHB to the anterior part of the tectum. MHB: Midbrain Hindbrain boundary, HB: hindbrain, A: anterior, P: posterior.

**Supplementary Figure S2 Eng2 mRNA measurement by quantitative RT-PCR.**

**
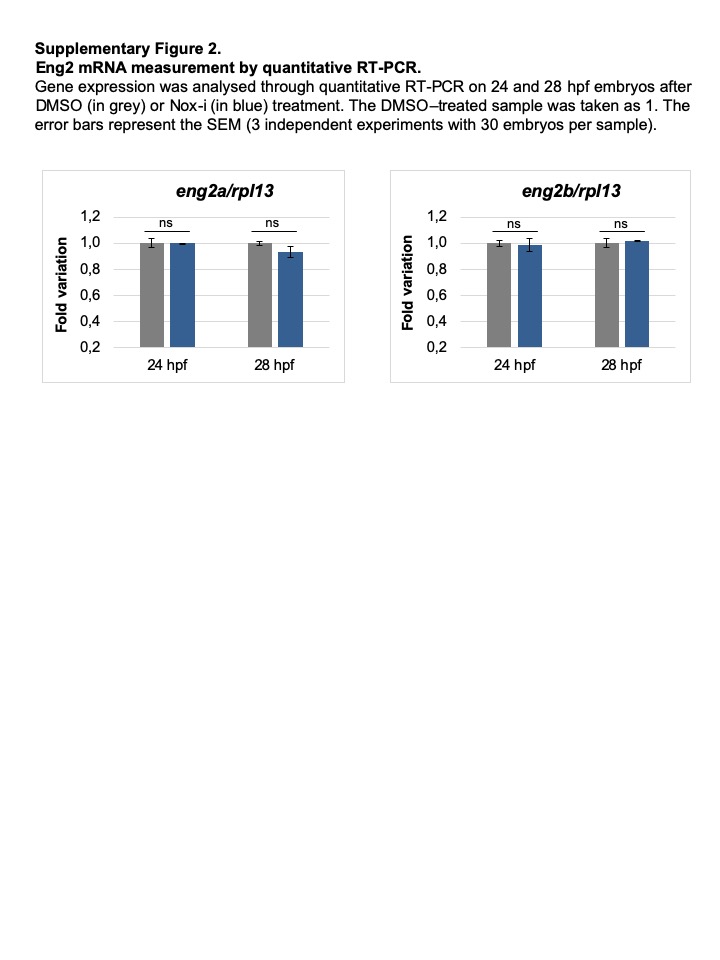
**

Gene expression was analysed through quantitative RT-PCR on 24 and 28 hpf embryos after DMSO (in grey) or Nox-i (in blue) treatment. The DMSO–treated samples were taken as 1. The error bars represent the SEM (3 independent experiments with 30 embryos per sample).

**Supplementary Figure S3. Manipulation of the H_2_O_2_ levels in cell culture.**


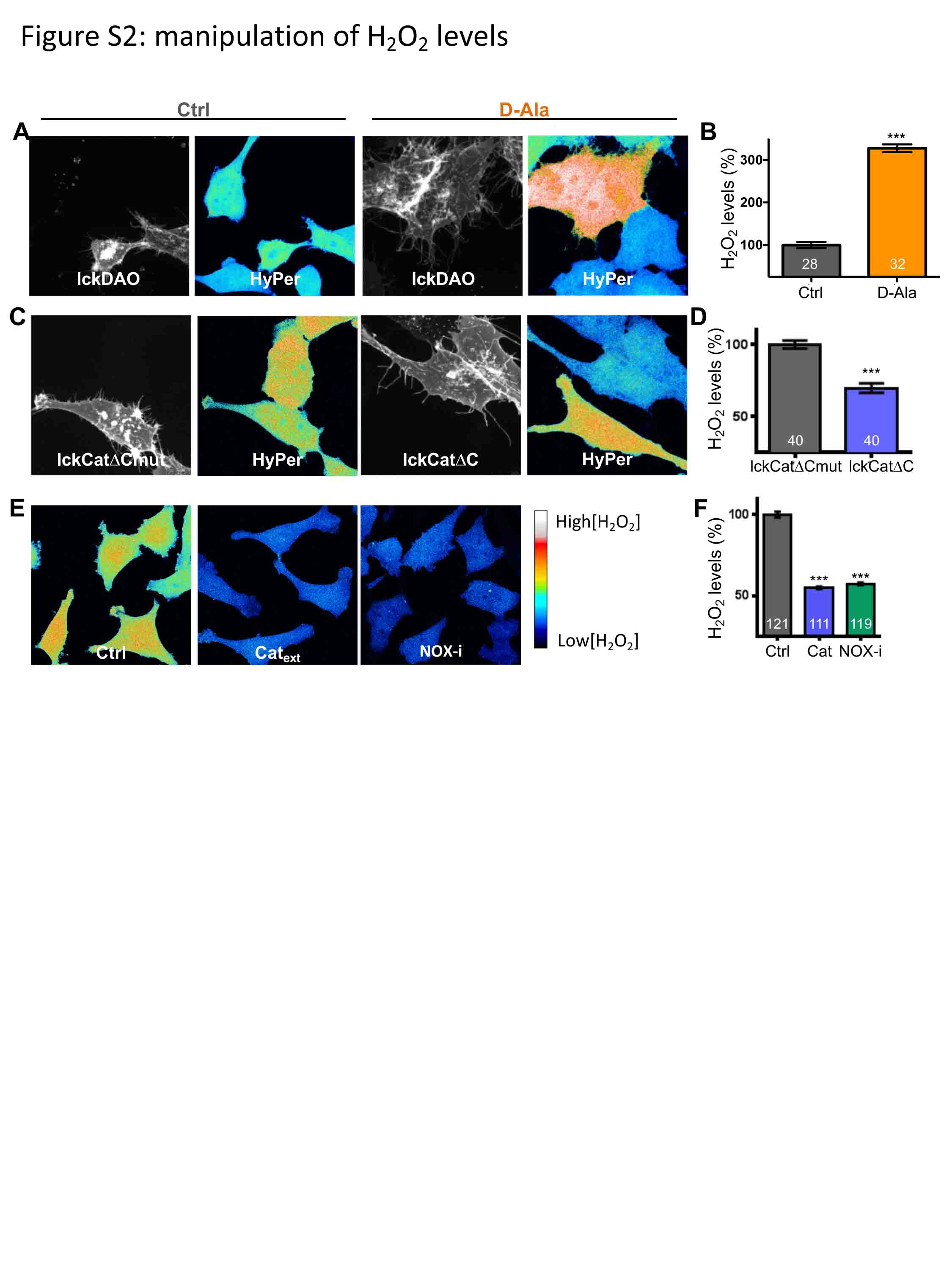


The stable HeLa-HyPer cells in which HyPer expression was controlled by doxycycline**^1^** were used to quantify the H_2_O_2_ levels upon genetic or pharmaceutical manipulation. The H_2_O_2_ levels were inferred from the YFP_500_/YFP_420_ excitation ratio of HyPer. (**A, B**) Cells were transfected with a plasmid coding membrane-associated DAO (Lck-mCherry-DAO) and incubated with or without D-alanine (10 mM). (**C, D**) Cells were transfected with a plasmid coding for active or inactive catalase (Lck-mCherry-Cat_ΔC_ and Lck-mCherry-CatΔ_Cmut_, respectively). (**E, F**) Purified catalase (Cat_ext_, 400 U/mL and/or NOX-i (10 μM) was added to the cell medium.

**Supplementary Figure S4. EN2 internalization under different redox conditions.**


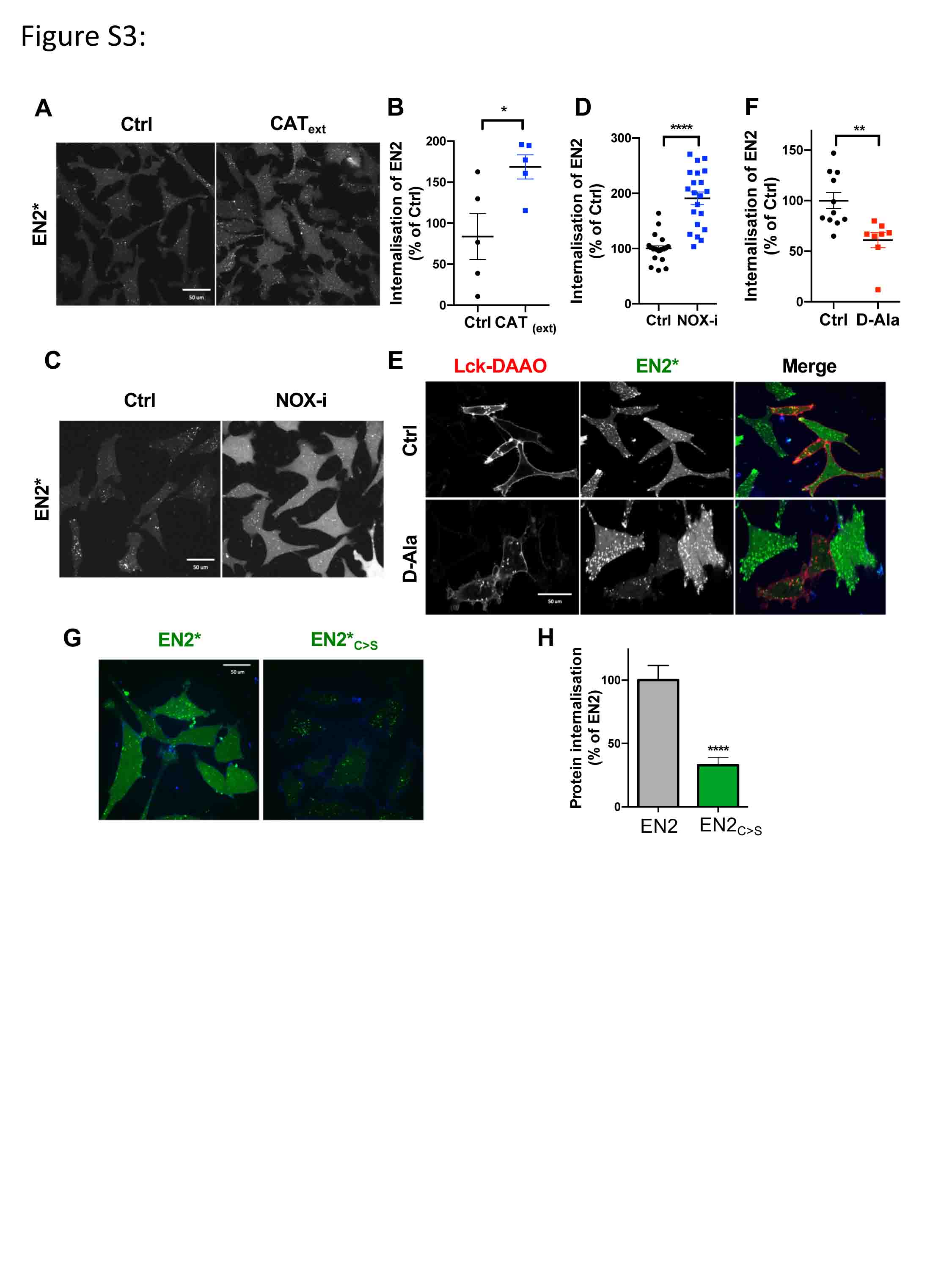


(**A**) Intracellular EN2-FITC (EN2*) staining in live cells following 30 min incubation in the presence (CAT_ext_) or absence (Ctrl) of extracellular catalase. (**B)** Quantification of internalization in the cells shown in A, p = 0,0278. (**C**) Intracellular EN2-FITC (EN2*) staining in live cells following 30 min incubation in the presence (NOX-i) or absence (Ctrl) of NOX-i (10 μM). (**D**) Quantification of the internalization in the cells shown in C, p < 0,0001. (**E**) Intracellular EN2-FITC (EN2*) staining in live cells transfected with LckDAO following 30 min incubation in the presence (D-Ala) or absence (Ctrl) of D-alanine (10 mM). (**F**) Quantification of the internalization in the cells shown in E, p = 0,0033. (**G**) Intracellular EN2-FITC (EN2*) or EN2_C>S_-FITC (EN2*_C>S_) staining in live cells following 30 min incubation (**H**) Quantification of the internalization in the cells shown in G, p < 0,0001.

**Supplementary Figure S5. EN2 spontaneously dimerizes and can be forced into a redox-insensitive dimer**.

(**A**) Purified recombinant EN2 and EN2_C>S_ proteins were analyzed on SDS PAGE in reducing (+DTT) or non-reducing (-DTT) conditions. (**B**) The crosslinked recombinant EN2 protein was analyzed on SDS PAGE in reducing (+DTT) or non-reducing (-DTT) conditions.

**Supplementary Table S1: Spatio-temporal variation of H_2_O_2_ and Eng levels during midbrain patterning.**

MHB: Midbrain Hindbrain Boundary; Eng tot: total Eng signal; Eng nuc: nuclear Eng signal.

|  |  | MHB | Tectum | |
| --- | --- | --- | --- | --- |
|  |  |  | posterior | anterior |
| 24 hpf | H_2_O_2_ | + | - | - |
|  | Eng tot | ++ | + | +/- |
|  | Eng nuc | +++ | ++ | +/- |
| 26 hpf | H_2_O_2_ | ++ | ++ | + |
|  | Eng tot | + | + | +/- |
|  | Eng nuc | +++ | ++ | ++ |
| 28 hpf | H_2_O_2_ | +++ | +++ | ++ |
|  | Eng tot | + | + | +/- |
|  | Eng nuc | ++ | ++ | +/- |

**Supplementary Table S2: Plasmids used in this study**

**Brief description:**

Plasmids #458, #459, #460, #499, #1067 and #1094 were used for bacterial expression and purification of EN2 (#458), EN2_W>K_ (#459), EN2_C>S_ (#460), Pbx1A (#499), HiBiT-En2 (#1067) and HiBiT-EN2_C>S_ (#1094). Addition of the two last proteins to HeLa cells stably expressing LgBiT (Table S3) allows internalization quantification.

Plasmids #299, #456, #457 and #1125 were in vitro transcribed to give ubiquitous expression in zebrafish embryos after injection of mRNA coding for EN2-ER^T2^ (#299), EN2_W>K_-ER^T2^ (#456), EN2_C>S_-ER^T2^ (#457) and HyPer7 (#1125).

Plasmids #369, #444, #447, #960, #962, #964 and #966, were transfected in HeLa cells as transcriptional reporter (#369) or expressors of EN2 (#444) or EN2_C>S_ (#447), or to express various redox regulators: DAO (#960), membrane-bound active or inactive Catalase (respectively #962 and #964) or membrane-bound DAO (#966).

The transRUSH plasmids #1163, #1164 and #1165 allow bi-directional expression of a common transmembrane hook-and-sensor protein (cytosolic hook and extracellular sensor), and of various membrane-bound redox regulators in the opposite direction: DAO (#1163), active or inactive Catalase (respectively #1164 and #1165). Their transfection in HeLa cells stably expressing doubly-tagged En2 (SBP-myc-EN2-HiBiT and SBP-myc-EN2_C>S_-HiBiT, Table S2) allows secretion quantification.

Plasmids #1185, #1205 and #1231 enable embryonic expression of the 4F11 scFv and mCherry in the Eng2a expression territory.

Supplementary **Table S3.**

Stable HeLa cell lines used in this study (in the order they appear in the figures)

| Name | Construct used for HeLa Flp-In | Expressed protein  (cf abbreviations) |
| --- | --- | --- |
| HyPer | pcDNA7Hyper | HyPer |
| transRUSH-EN2 | pcDNA7SBPmycEN2LVBi | SBP-myc-EN2-HiBiT |
| GBi | pcDNA7LGBi | LgBiT |
| transRUSH-EN2C>S | pcDNA7SBPmycEN2CLVBi | SBP-myc-EN2_C>S_-HiBiT |
| LckHyPer | pcDNA7LckHyPer | Lck-HyPer |

All stable cell lines were prepared using the HeLa Flp-In cell line kindly provided by Stephen Taylor**^2^**

**Abbreviations used in Supplementary Tables S2 and S3** (in alphabetical order)

4F11scFv: single-chain antibody recognizing EnG2 proteins

Cat_ΔC_: active Catalase without a lysosomal targeting signal (from Addgene plasmid #67635)

Cat_ΔCmut_: inactive form of Cat_ΔC_ (aa 64-78 substituted with SGPSGIPGGVVGAFP)

CMV/tetO: human CMV enhancer and promoter including 2 Tetracycline operators

CMVmin: minimal promoter from human CMV

DAO: D-amino acid-oxidase

EN2_C>S_: EN2 (C175S)

EN2_W>K_: EN2 (W169K,W172K)

Eng2a(-5.0, 4.5): minimal enhancer driving expression at MHB**^3^**

Gal4BD-FF: DNA-binding domain of Gal4 fused to dimerized minimal activation domain F

HiBiT: small portion of the split NanoBit

hisCTagSci: his6-Cherry^TM^Tag(Delphigenetics)-PreScission cleavage site

Igk: signal peptide of IgK

LgBiT: large portion of the split NanoBit

Lck: myristoylation-palmitoylation signal of Lck (aa 1-0)

myc: 10-aa tag taken from c-myc

NanoLuc: Nanoluciferase

P2A: skipping peptide from porcine teschovirus-1.

SBP: streptavidin-binding protein

sCMV: simian CMV enhancer and promoter

SiL: IL2 signal peptide

STRP: core streptavidin

Tm1: *B. Taurus* CD44 (aa 186-299), including the transmembrane domain

**Supplementary Figure Table S4.**

**Sample Size:**

| Figure | condition | Sample size | Mean | Std. Deviation | Std. Error of Mean | P-value | Confidence interval |
| --- | --- | --- | --- | --- | --- | --- | --- |
| **Figure 1b**  **Figure 1c** |  |  |  |  |  |  |  |
|  | 24 hpf | 8 |  |  |  |  |  |
|  | 26 hpf | 12 |  |  |  |  |  |
|  | 28 hpf | 13 |  |  |  |  |  |
| **Figure 1e** |  | 3 |  |  |  |  |  |
| **Figure 1f** |  |  |  |  |  |  |  |
|  | Ctrl | 8 | 100 | 3,713 | 1,313 | 0.0271 | 0,95 |
|  | NOX-i | 6 | 93,63 | 5,785 | 2,362 |  |  |
| **Figure 1g** |  |  |  |  |  |  |  |
|  | Ctrl | 8 |  |  |  | 0,0001<p-value<0,05 from 0 to 64 µm | 0,95 |
|  | NOX-i | 14 |  |  |  |  |  |
| **Figure 1h** |  |  |  |  |  |  |  |
|  | Ctrl | 5 |  |  |  | 0,0001<p-value<0,05 from 0 to 25 µm | 0,95 |
|  | NOX-i | 4 |  |  |  |  |  |
| **Figure 2b** |  |  |  |  |  |  |  |
|  | Ctrl | 24 | 100 | 15,2554221 | 3,114 | < 0,0001 | 0,95 |
|  | D-Ala | 24 | 143,8 | 26,0870658 | 5,325 |  |  |
| **Figure 2c** |  |  |  |  |  |  |  |
|  | CAT_mut_ | 10 | 100 | 31,2116805 | 9,87 | < 0,0001 | 0,95 |
|  | CAT | 10 | 24,95 | 31,2749261 | 9,89 |  |  |
| **Figure 2d** |  |  |  |  |  |  |  |
|  | Ctrl | 7 | 100 | 22,3565986 | 8,45 | < 0,0001 | 0,95 |
|  | CAT_ext_ | 7 | 43,43 | 11,4137712 | 4,314 |  |  |
| **Figure 2e** |  |  |  |  |  |  |  |
|  | Ctrl | 10 | 100 | 28,871595 | 9,13 | < 0,0001 | 0,95 |
|  | NOX-i | 10 | 20,7 | 14,5085299 | 4,588 |  |  |
| **Figure 2g** |  |  |  |  |  |  |  |
|  | Ctrl | 6 | 100 | 31,9658411 | 13,05 | 0,0155 | 0,95 |
|  | D-Ala | 6 | 57,33 | 15,6424415 | 6,386 |  |  |
| **Figure 2h** |  |  |  |  |  |  |  |
|  | Ctrl | 5 | 100 | 17,5576058 | 7,852 | 0,0007 | 0,95 |
|  | CAT_ext_ | 5 | 189,2 | 29,1136051 | 13,02 |  |  |
| **Figure 2i** |  |  |  |  |  |  |  |
|  | Ctrl | 8 | 100 | 10,8498465 | 3,836 | 0,005 | 0,95 |
|  | NOX-i | 8 | 171,3 | 50,7702669 | 17,95 |  |  |
| **Figure 3c** |  |  |  |  |  |  |  |
| **A** | Ctrl | 3 | 0,2726 | 0,03346322 | 0,01932 | A vs B  and A vs C  p< 0,0001 | 0,95 |
| **B** | EN2 | 3 | 1,82 | 0,07106604 | 0,04103 |  |  |
| **C** | EN2_C>S_ | 3 | 2,434 | 0,1473802 | 0,08509 |  |  |
|  |  |  |  |  |  |  |  |
| Figure | condition | Sample size | Mean | Std. Deviation | Std. Error of Mean | P-value | Confidence interval |
| **Figure 3d** |  |  |  |  |  |  |  |
|  | EN2 | 8 | 100 | 21,190576 | 7,492 | < 0,0001 | 0,95 |
|  | EN2_C>S_ | 8 | 47,88 | 11,9444477 | 4,223 |  |  |
| **Figure 3e** |  |  |  |  |  |  |  |
| **A** | Ctrl/EN2 | 4 | 100 | 5,228 | 2,614 | A vs B  p< 0,0001  B vs C p=0,2193 | 0,95 |
| **B** | Ctrl/EN2_C>S_ | 4 | 45,33 | 5,812 | 2,906 |  |  |
| **C** | D-Ala/EN2_C>S_ | 4 | 37,33 | 9,334 | 4,667 |  |  |
| **Figure 3f** |  |  |  |  |  |  |  |
| **A** | CAT_mut_/EN2 | 4 | 100 | 25,32 | 12,66 | A vs B p=0,0070 B vs C p=0,0945 | 0,95 |
| **B** | CAT_mut_/ EN2_C>S_ | 4 | 50 | 11,506 | 5,753 |  |  |
| **C** | CAT/ EN2_C>S_ | 4 | 69,75 | 17,428 | 8,714 |  |  |
| **Figure 3g** |  |  |  |  |  |  |  |
|  | EN2 | 10 | 100 | 21,29794 | 6,735 | < 0,0001 | 0,95 |
|  | EN2_C>S_ | 10 | 51,96 | 5,30946419 | 1,679 |  |  |
| **Figure 3h** |  |  |  |  |  |  |  |
| **A** | Ctrl/EN2 | 5 | 100 | 33,8540692 | 15,14 | A vs B p=0,0116 C vs D p=0,3728 | 0,95 |
| **B** | DTT/EN2 | 6 | 34,83 | 6,14576976 | 2,509 |  |  |
| **C** | Ctrl/ EN2_C>S_ | 4 | 45,25 | 9,912 | 4,956 |  |  |
| **D** | DTT/ EN2_C>S_ | 4 | 50,5 | 2,646 | 1,323 |  |  |
| **Figure 3i** |  |  |  |  |  |  |  |
|  | EN2 | 6 | 100 | 7,77468044 | 3,174 | < 0,0001 | 0,95 |
|  | EN2_C>S_ | 6 | 187 | 33,435535 | 13,65 |  |  |
| **Figure 4b** |  |  |  |  |  |  |  |
| **A** | Ctrl | 23 | 100 | 33,8777539 | 7,064 | A vs B  p< 0,0001 A vs C p=0,8472 A vs D p=0,2 B vs C p<0,0001 B vs D  p< 0,0001 | 0,95 |
| **B** | EN2 | 19 | 173,6 | 54,2247029 | 12,44 |  |  |
| **C** | EN2_W>K_ | 29 | 97,87 | 29,9738273 | 5,566 |  |  |
| **D** | EN2_C>S_ | 18 | 86,99 | 26,0667844 | 6,144 |  |  |
| **Figure 4d** |  |  |  |  |  |  |  |
| **A** | Ctrl | 39 | 100 | 40,648692 | 6,509 | A vs B  p< 0,0001  A vs C p=0,1610 B vs C p=0,0088 | 0,95 |
| **B** | EN2 | 24 | 168 | 51,7822132 | 10,57 |  |  |
| **C** | EN2 4G11 | 16 | 120 | 57 | 14,25 |  |  |
| **Figure 4f** |  |  |  |  |  |  |  |
|  | Ctrl | 11 | 100 | 14,561047 | 4,39032085 | 0,0001 | 0,95 |
|  | scFv4F11 | 18 | 81,2842604 | 8,08374838 | 1,90535777 |  |  |
|  |  |  |  |  |  |  |  |

**References**

1 Gauron, C. *et al.* Hydrogen peroxide (H2O2) controls axon pathfinding during zebrafish development. *Dev Biol* **414**, 133-141 (2016).

2 Tighe, A., Staples, O. & Taylor, S. Mps1 kinase activity restrains anaphase during an unperturbed mitosis and targets Mad2 to kinetochores. *J Cell Biol* **181**, 893-901, doi:10.1083/jcb.200712028 (2008).

3 Maurya, A. K. *et al.* Integration of Hedgehog and BMP signalling by the engrailed2a gene in the zebrafish myotome. *Development* **138**, 755-765, doi:10.1242/dev.062521 (2011).
