# Supplementary material for: H2O2 and Engrailed 2 paracrine activity synergize to shape the zebrafish optic tectum
Source: Commun Biol. 2020 Sep 29;3:536. doi: 10.1038/s42003-020-01268-7 (PMC7524761; doi:10.1038/s42003-020-01268-7)
Supplement: Supplementary file 2 — Description of Additional Supplementary Files [file 42003_2020_1268_MOESM2_ESM.pdf]

Description of Additional Supplementary Files

File Name: Supplementary Data 1
Description: Source data behind graphs
