# Supplementary material for: H2O2 and Engrailed 2 paracrine activity synergize to shape the zebrafish optic tectum
Source: Commun Biol. 2020 Sep 29;3:536. doi: 10.1038/s42003-020-01268-7 (PMC7524761; doi:10.1038/s42003-020-01268-7)
Supplement: Supplementary file 4 — Reporting Summary [file 42003_2020_1268_MOESM4_ESM.pdf]

nature research

Corresponding author(s): Sophie Vriz

Last updated by author(s): Aug 21, 2020

Reporting Summary

Nature Research wishes to improve the reproducibility of the work that we publish. This form provides structure for consistency and transparency in reporting. For further information on Nature Research policies, seeAuthors & Referees and theEditorial Policy Checklist .

Statistics

For all statistical analyses, confirm that the following items are present in the figure legend, table legend, main text, or Methods section.

n/a	Confirmed	
☐	☒	The exact sample size (*n*) for each experimental group/condition, given as a discrete number and unit of measurement
☐	☒	A statement on whether measurements were taken from distinct samples or whether the same sample was measured repeatedly
☐	☒	The statistical test(s) used AND whether they are one- or two-sided<br>*Only common tests should be described solely by name; describe more complex techniques in the Methods section.*
☒	☐	A description of all covariates tested
☒	☐	A description of any assumptions or corrections, such as tests of normality and adjustment for multiple comparisons
☐	☒	A full description of the statistical parameters including central tendency (e.g. means) or other basic estimates (e.g. regression coefficient) AND variation (e.g. standard deviation) or associated estimates of uncertainty (e.g. confidence intervals)
☐	☒	For null hypothesis testing, the test statistic (e.g. *F*, *t*, *r*) with confidence intervals, effect sizes, degrees of freedom and *P* value noted<br>*Give P values as exact values whenever suitable.*
☒	☐	For Bayesian analysis, information on the choice of priors and Markov chain Monte Carlo settings
☒	☐	For hierarchical and complex designs, identification of the appropriate level for tests and full reporting of outcomes
☒	☐	Estimates of effect sizes (e.g. Cohen's *d*, Pearson's *r*), indicating how they were calculated

Our web collection on statistics for biologists contains articles on many of the points above.

Software and code

Policy information about availability of computer code

Data collection	n.a.
Data analysis	FIJI (ImageJ)<br>Excell (Microsoft)<br>PRISM8 (GraphPad)

For manuscripts utilizing custom algorithms or software that are central to the research but not yet described in published literature, software must be made available to editors/reviewers. We strongly encourage code deposition in a community repository (e.g. GitHub). See the Nature Research guidelines for submitting code & software for further information.

Data

Policy information about availability of data

All manuscripts must include a data availability statement. This statement should provide the following information, where applicable:

- Accession codes, unique identifiers, or web links for publicly available datasets
- A list of figures that have associated raw data
- A description of any restrictions on data availability

Source data are available in Supplementary Data 1. All other data underlying the findings of the study are available from the corresponding author upon reasonable request.

Field-specific reporting

Please select the one below that is the best fit for your research. If you are not sure, read the appropriate sections before making your selection.

Life sciences study design

All studies must disclose on these points even when the disclosure is negative.

Sample size	No statistical methods were used to predetermine sample size
Data exclusions	No data exclusion. For imaging, embryos were mounted in agarose. Animals out of focus were discarded.
Replication	All the findings has been replicated
Randomization	Animals were picked from a pool of animals.
Blinding	The investigators were not blinded to group allocation during data collection and analysis

Reporting for specific materials, systems and methods

We require information from authors about some types of materials, experimental systems and methods used in many studies. Here, indicate whether each material, system or method listed is relevant to your study. If you are not sure if a list item applies to your research, read the appropriate section before selecting a response.

Materials & experimental systems

n/a	Involved in the study	
☐	☒	Antibodies
☐	☒	Eukaryotic cell lines
☒	☐	Palaeontology
☐	☒	Animals and other organisms
☒	☐	Human research participants
☒	☐	Clinical data

Methods

n/a	Involved in the study	
☒	☐	ChIP-seq
☒	☐	Flow cytometry
☒	☐	MRI-based neuroimaging

Antibodies

Antibodies used	4D9 anti engrailed antibody was deposited to DSHB by Goodman C, and used at a 1:500 dilution,
Validation	Patel et al. (1989) Cell 58, 955-968

Eukaryotic cell lines

Policy information about cell lines

Cell line source(s)	HeLa Flp-In cell line provided by Dr Stephen Taylor
Authentication	Tighe et al (2008) J. Cell Biol 181, 893-901
Mycoplasma contamination	This cell line was tested negative for mycoplasma contamination
Commonly misidentified lines (See ICLAC register)	n.a.

Animals and other organisms

Policy information about studies involving animals; ARRIVE guidelines recommended for reporting animal research

Laboratory animals	zebrafish
Wild animals	This investigation do not involved wild animals
Field-collected samples	This investigation do not involved field-collected samples
Ethics oversight	French Ministry of Agriculture (n°C75-05-12)

Note that full information on the approval of the study protocol must also be provided in the manuscript.
